# Health-related quality of life measured by SF-36 among postdelivery mothers attending maternal and child health clinic in Eastern Nepal: a cross-sectional study

**DOI:** 10.1097/MS9.0000000000000439

**Published:** 2023-04-04

**Authors:** Pratiksha Chapagain, Prajjwal Pyakurel, Ayush Anand, Ashwini Gupta, Durga Subedi

**Affiliations:** a Institute of Medicine, Tribhuvan University, Maharajgunj Nursing Campus, Kathmandu; b School of Public Health and Community Medicine; c B.P. Koirala Institute of Health Sciences, Dharan, Nepal

**Keywords:** cross-sectional study, mothers, Nepal, postpartum period, quality of life

## Abstract

Assessing health-related quality of life (HRQoL) and its associated factors is essential for providing adequate healthcare and developing necessary interventions in women postdelivery. This study intended to find out the HRQoL score and related factors among women postdelivery in Nepal.

**Methodology:**

This was a cross-sectional study using nonprobability sampling conducted at a Maternal and Child Health (MCH) Clinic in Nepal. The study participants were 129 women postdelivery to 12 months who visited the MCH Clinic from 2 September 2018 to 28 September 2018. Outcome measures were sociodemographic, clinical indicators, obstetric indicators, and their relation with the overall HRQoL score of postdelivery mothers using the Short Form Health Survey (SF-36) Version 1.

**Results:**

Of 129 respondents, 68.22% were in the 21–30 age group, 36.43% were upper caste, 88.37% were Hindu, 87.60% were literate, 81.39% were homemakers, 53.49% with income less than 12 months, 88.37% had family support, and 50.39% with vaginal deliveries. HRQoL was significantly more in employed women (*P*=0.037), those with family support (*P*=0.003), and those who had a cesarean section (*P*=0.02) and wanted pregnancy (*P*=0.040).

**Conclusion:**

HRQoL in women postdelivery can be influenced by employment status, family support, type of delivery, and desirability of pregnancy.

## Introduction

Highlights

- Health-related quality of life in women postdelivery can be influenced by employment status, family support, delivery type, and pregnancy desirability.
- This study provides baseline data for future studies on the quality of life in women postdelivery in Nepal.

WHO defined quality of life (QoL) as ‘an individual’s perception of their position in life in the context of the culture and value systems in which they live and in relation to their goals, expectations, standards, and concerns’^1^. QoL is a broad concept, and nowadays, the term health-related quality of life (HRQoL) is employed, which can be defined as a subjective understanding regarding one’s own physical and mental health over time^2^. HRQoL can assess people’s self-reported health issues and devise interventions to address those issues at the individual, population, and policy-making levels^3^.

After childbirth, the body undergoes many physical, physiological, and psychological changes^4^–^6^. In this phase, women are vulnerable to various health-related issues and disorders. Psychiatric disorders such as postpartum depression, anxiety, and rarely psychosis can occur^7^,^8^. Other conditions, such as postpartum hemorrhage and cardiomyopathy, can also occur^9^,^10^. These conditions can negatively affect the health of the mother and child. Hence, it is crucial to assess the HRQoL to provide the best care and necessary interventions.

Various sociodemographic and obstetric factors can contribute to the HRQoL in women postdelivery. Nepal’s National Demographic and Health Survey (NDHS) in 2016 showed that ∼41% of deliveries were done at home without assistance from trained healthcare workers^11^. Also, the survey revealed a big socioeconomic divide; only 34% of women in the lower quintile and only 38% without formal education underwent institutional delivery^11^. The scenario was similar regarding postnatal care, with only 37% of women in the low quintile availing of postnatal care compared to 81% of women in the high quintile^11^. Moreover, cesarean section deliveries were more likely in private facilities, urban areas, and among women with formal education^11^. In addition, women in low-quantile, rural areas and without formal education were more likely to have problems accessing health care^11^. These barriers may lead to poor QoL in women.

Jeong *et al.* 12 found marital intimacy as the most crucial element influencing the QoL in women postdelivery. Another study showed an association between relationship satisfaction and higher HRQoL scores^13^. From these studies, we can understand the importance of a woman’s relationship with their spouse and its association with HRQoL. Also, occupational status can significantly influence the HRQoL of a mother^12^. Studies have shown that employed women had higher psychological and environmental HRQoL postpartum^14^,^15^. However, Malaju *et al*.^16^ reported that postpartum women who worked outside their homes had poorer HRQoL. Other studies did not find any significant association of HRQoL with different modes of delivery^17^,^18^, level of education^15^,^19^, and income level^15^,^19^. Sadat *et al*.^20^ reported that vaginal deliveries were associated with better HRQoL than cesarean section deliveries. In contrast, Malaju *et al*.^16^ reported that women with vaginal deliveries were likelier to have a poorer HRQoL. Also, lower income was associated with poorer HRQoL after childbirth^21^,^22^. Level of education also affects the HRQoL^16^,^23^, with lower education levels in women significantly associated with a lower HRQoL^16^. In contrast, some studies have reported that higher education was significantly associated with a lower HRQoL^14^,^24^.

Earlier studies have found various associated factors of HRQoL among women postdelivery. However, there were discrepancies in the findings of various studies, which varied in different settings. Also, the NDHS 2016 showed that women with low socioeconomic status faced difficulty accessing maternal healthcare services. In addition, no investigation was conducted in Nepal using the SF-36 questionnaire. All the factors revealed a need to study the HRQoL in women postdelivery. Hence, we aim to assess the HRQoL and its associated factors among women postdelivery in Nepal.

## Materials and methods

### Study design

This cross-sectional study involved women postdelivery to 12 months who visited the Maternal and Child Health (MCH) Clinic in Nepal.

### Setting

Nepal has 125 government hospitals under the Ministry of Health and Population, with 18 government hospitals in Province 1^17^,^25^. The study site is one of the oldest government hospitals in Province 1, providing preventive, curative, and promotive services to the public of eastern Nepal. The MCH Clinic of our selected study site caters to immunization, family planning, and abortion services in the eastern region of Nepal.

### Study population

The study population included the mothers who attended the MCH clinic from postdelivery to 12 months postdelivery. Mothers with psychiatric disorders were excluded from the study population.

### Sample size and sampling technique

We used a nonprobability purposive sampling technique to select mothers meeting the inclusion criteria. The sample size was calculated at a 95% CI with a 5% allowable error based on an SD of 27.71 by using the formula $n=z^{2}\sigma^{2}/d^{2}$ 17,

where *n* is the required sample size; *z* is the value of standard normal variate at the desired level of confidence; *σ* is the variance; and *d* is the allowable error.

Hence, $n\;=z^{2}\sigma^{2}/d^{2}\;=\;\frac{{\left(1.96\right)}^{2\;}\;\times \;{\left(27.71\right)}^{2}}{{\left(5\right)}^{2}}\;=118$ .

We considered a nonresponse rate of 10%. Hence, the total sample size was 129.

### Questionnaire design

We used Short Form Health Survey (SF-36) Version 1.0 to assess study participants’ HRQoL after obtaining permission for Research and Development (RAND)^26^. RAND SF-36 was already validated for use in Nepal by Bhandari *et al*.^27^. However, we again pretested the translated Nepali SF-36 among 13 mothers in another MCH Clinic in Nepal. Cronbach’s *α* was used to test the reliability, and the reported value was 0.752. Patient participants were involved in the study’s pretesting. However, there was no public involvement in our research. The final questionnaire included three sections.

#### Section 1: Sociodemographic information

This part included questions about the mother’s age, marital status, educational level, ethnicity, religion, occupation, family income, type of family, and support. Ethnicity was classified based on the NDHS 2016 survey of Nepal^11^. To better understand socioeconomic status, upper caste, and relatively advantaged Janajati were combined as a privileged group. Similarly, Dalit, disadvantaged non-Dalit terai, religious minorities, and disadvantaged Janajati were categorized as underprivileged. Categorizing income level was done based on the study by Mishra *et al*.^28^.

#### Section 2: Questions related to obstetric variables

This part included obstetric variables, including parity, number of living children, place of delivery, mode of delivery, postpartum duration, the desirability of pregnancy, and maternal complications.

#### Section 3: Questions related to the QoL of postnatal mothers (SF-36 questionnaire-standard tool)

This part consisted of the RAND SF-36 version 1.0 questionnaire tool with 36 questions translated into various languages^26^. It measures eight health-related concepts: physical functioning, role limitation due to physical problems (role-physical), bodily pain, general health perceptions, vitality (energy and fatigue), social functioning, role limitation due to emotional problems (role-emotional), and perceived mental health/emotional well-being^26^. QoL is the composite summary of two domains Physical Health-Related Quality of Life (PQoL)/Physical Component Summary (PCS) and Mental Health-Related Quality of Life (MQoL)/Mental Component Summary (MCS)^26^. PQoL includes physical functioning, role-physical, bodily pain, and general health^29^. MQoL includes vitality, social functioning, role-emotional and mental health^29^.

The scores on each subscale range from 0 to 100^30^. A zero score indicates the lowest level of health measurement on the scale, and higher scores indicate better HRQoL. The questions were combined and weighted on two scales, that is PCS and MCS^29^. The mean for each SF-36 subscale was classified into poor HRQoL level (with a score of 0–49) and a good level of HRQoL (with a score of 50–100)^30^.

### Variables studied

Independent variables: age of mother, education status, ethnicity, religion, occupation, family income, type of family, family support, parity, number of living children, the desirability of pregnancy, mode of delivery, postpartum duration, place of delivery, and complications in recent delivery.

#### Dependent variable

Overall health-related QoL score of postdelivery mothers.

### Data collection

**Figure 1:**
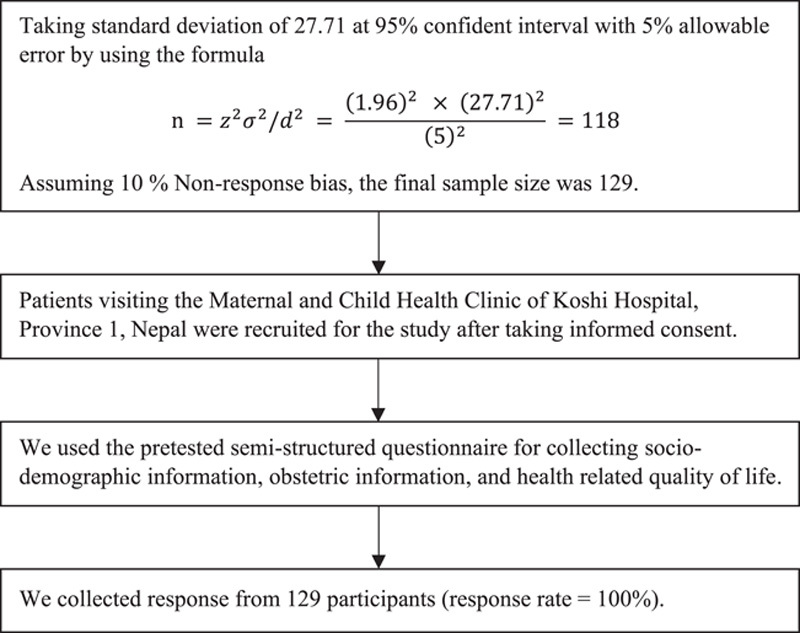
Enrollment procedure of study participants.

Data was collected through face-to-face interviews using the RAND SF-36 Nepali version questionnaire from 2 September 2018 to 28 September 2018. We collected a total of 129 responses (Fig. 1).

### Statistical analysis

The collected data were entered into an excel spreadsheet, followed by data cleaning. Then the data were exported into Statistical Package for Social Sciences (SPSS) software version 16 for analysis. Data analysis was done by using descriptive and inferential statistics. Descriptive statistics, that is number, percentage, mean, and standard deviation, were used to describe the demographic and obstetric variables. Mann–Whitney *U* test was applied to compare postnatal mothers’ QoL in different delivery modes. Similarly, Kruskal–Wallis *H* and Mann–Whitney *U* tests were used to examine the difference between the QoL of postnatal mothers with selected sociodemographic and obstetric variables. Statistical significance was considered with a *P* value 0.05 or less. This work has been reported in line with STROCSS (strengthening the reporting of cohort, cross-sectional and case–control studies in surgery) 2021 criteria^31^.

## Results

### Sociodemographic characteristics

Of 129 respondents, more than two-thirds (88, 68.22%) were in the 21–30 age group (Table 1). All the participants were married, and the majority (113, 87.60%) were literate. Approximately 36.43% (47) of the respondents belonged to the upper caste, 26.36% (34) were disadvantaged Janajati, and 21.70% (28) belonged to the disadvantaged non-Dalit terai caste. And the majority (114, 88.37%) of the study participants were Hindu. Regarding occupation, most (105, 81.39%) were homemakers. Nearly one-fifth (24, 18.61%) of the respondents had a family income not sufficient for 6 months of living costs; two-thirds (81, 62.79%) were living in a joint family, and the majority (114, 88.37%) received family support.

**Table 1:** Sociodemographic characteristics of the respondents (*n*=129).

Variables	Frequency (*n*)	Percentage (%)
Age		
≤20 years	25	19.38
21–30 years	88	68.22
31–40 years	16	12.40
Mean age±SD=25.14±4.45 years		
Educational status		
Illiterate	16	12.40
Primary	19	14.73
Secondary	38	29.46
Higher secondary	25	19.38
Bachelor and above	31	24.03
Ethnicity		
Upper caste	47	36.43
Disadvantaged Janajati	34	26.36
Disadvantaged non-Dalit terai caste group	28	21.70
Religious minorities	7	5.43
Relatively advantaged Janajati	7	5.43
Dalit	6	4.65
Religion		
Hinduism	114	88.37
Islamic	10	7.75
Buddhism	4	3.10
Christianity	1	0.78
Occupation		
Homemaker	105	81.39
Nongovernment employee	13	10.08
Self-employ	9	6.98
Government employee	2	1.55
Family income (annual)		
Income enough for less than 6 months	24	18.61
Income enough for 6–12 months	45	34.88
Income enough for 12 months and surplus	60	46.51
Family type		
Joint	81	62.79
Nuclear	48	37.21
Family support		
Yes	114	88.37
No	15	11.63

### Obstetric characteristics

More than half (70, 54.26%) of the respondents were primiparous, and approximately three-fifths (60.46%) had only one living child (Table 2). Almost all (124, 96.12%) of the respondents had desirable pregnancies, and the majority (60, 46.51%) belonged from 6 weeks to 6 months postpartum. Almost all (124, 96.12%) women had institutional deliveries, nearly half (65, 50.39%) of the deliveries were vaginal, and the majority (118, 91.47%) delivered without complications.

**Table 2:** Obstetric characteristics of the respondents (*n*=129).

Variables	Frequency	Percentage (%)
Parity		
Primiparous	70	54.26
Multiparous	59	45.74
Number of living children		
One	78	60.46
Two	42	32.56
Three	9	6.98
Desirability of pregnancy		
Wanted	124	96.12
Unwanted	5	3.88
Duration of postpartum period		
Up to 6 weeks	25	19.38
>6 weeks to 6 months	60	46.51
>6 months	44	34.11
Place of delivery		
Health institution	124	96.12
Home	5	3.88
Mode of delivery		
Vaginal delivery	65	50.39
Cesarean section	64	49.61
Complications in recent delivery		
No	118	91.47
Yes	11	8.53
Complications (*n*=11)		
Increased blood pressure	6	54.55
Vaginal bleeding	5	45.45

### HRQoL

Table 3 shows the mean score of the QoL in different domains. In the physical QoL domain, the highest (74.65) mean score was observed in physical functioning, whereas the lowest (48.26) mean score was observed in role limitation due to physical health. For the mental QoL, the highest mean score (86.43) was observed in social functioning, whereas the lowest (48.58) mean score was observed in role limitation due to emotional health. The QoL in the mental component score was 64.48±14.18. Likewise, the physical component’s mean score was 64.09±15.49. The postnatal mothers’ overall QoL score was 64.28±12.87.

**Table 3:** Health-related quality of life (HRQoL) scores of the respondents (*n*=129).

Variables	HRQoL scores (mean±SD)
Physical quality of life (QoL)	
Physical Functioning (PF)	74.65±16.57
Role-Physical (RP)	48.26±40.41
Bodily Pain (BP)	71.25±22.31
General Health (GH)	62.21±15.59
Total Physical QoL	64.09±15.49
Mental QoL	
Role-Emotional (RE)	48.58±40.61
Vitality (VT)	54.61±15.95
Social Functioning (SF)	86.43±18.36
Mental Health (MH)	68.31±14.40
Total Mental QoL	64.48±14.18
Overall HRQoL	64.28±12.87

QoL score: 0–49 – poor QoL; 50–100 – good QoL.

### Sociodemographic and obstetric predictors of HRQoL

Employed women (*P*=0.037) and those with family support (*P*=0.003) had significantly higher QoL (Table 4). Women with cesarean section (*P*=0.020) and desirable pregnancies (*P*=0.040) deliveries had a significantly higher QoL in the mental health subscale than women with vaginal delivery (Table 5). Also, women with desirable pregnancies (*P*=0.040) had higher QoL than those with unwanted pregnancies (Table 6). We did not observe any statistically significant difference between the physical, mental, and overall QoL of respondents with different modes of delivery. Also, no statistically significant relation of QoL with parity, living children, duration of the postpartum period, place of delivery, and complications in recent delivery was found.

**Table 4:** Difference between health-related quality of life of the respondents with selected sociodemographic variables (*n*=129).

Variables	Frequency (%)	Total QoL score (mean±SD)	*P*
Age in years			
≤20 years	25 (19.4)	64.84±8.63	0.833^a^
21–30 years	88 (68.2)	63.65±13.48	
31–40 years	16 (12.4)	66.84±15.19	
Educational status			
Literate	113(87.60)	64.76±12.49	0.349^b^
Illiterate	16 (12.40)	60.98±15.29	
Ethnicity			
Privileged^c^	54 (41.86)	65.44±12.54	0.583^b^
Underprivileged^d^	75 (58.14)	63.46±13.12	
Occupation			
Homemaker	105 (81.40)	63.21±12.83	0.037* ^,b^
Employed	24 (18.60)	69.02±12.19	
Family income (annual)			
Income enough for less than 6 months	24 (18.6)	63.32±7.78	0.656^a^
Income enough for 6–12 months	45 (34.9)	65.31±13.65	
Income enough for 12 months and surplus	60 (46.5)	63.89±13.98	
Family type			
Nuclear	48 (37.21)	63.04±13.28	0.434^b^
Joint	81 (62.79)	65.03±12.64	
Family support			
Yes	114 (88.37)	65.74±11.77	0.003* ^,b^
No	15 (11.63)	53.29±15.80	

* *P*≤0.05 significant.

^a^ Kruskal–Wallis *H* test.

^b^ Mann–Whitney *U* test.

^c^ Upper caste, relatively advantageous Janajati.

^d^ Dalit, disadvantaged non-Dalit terai, religious minorities, and disadvantaged Janajati.

**Table 5:** Health-related quality of life (HRQoL) of the respondents in different modes of delivery (*n*=129).

	HRQoL score (mean±SD)		
Variables	Vaginal delivery (*n*=65)	Cesarean section (*n*=64)	*P*
Physical quality of life (QoL)			
Physical Functioning (PF)	77.38±15.18	71.88±17.56	0.109
Role-Physical (RP)	54.62±40.24	41.84±39.84	0.065
Bodily Pain (BP)	70.35±23.59	72.19±21.07	0.847
General Health (GH)	61.23±15.34	63.20±15.90	0.499
Total Physical QoL	65.89±15.88	62.27±14.98	0.113
Mental QoL			
Role-Emotional (RE)	53.33±42.41	43.75±38.43	0.200
Vitality (VT)	53.95±14.66	55.16±17.41	0.434
Social Functioning (SF)	86.54±17.72	86.33±19.12	0.884
Mental Health (MH)	65.42±13.27	71.25±14.99	0.020*
Total Mental QoL	64.84±14.97	64.12±13.45	0.876
Overall HRQoL	65.37±13.22	63.19±12.50	0.194

* *P*≤0.05 significant, Mann–Whitney *U* test.

**Table 6:** Difference between health-related quality of life (HRQoL) of the respondents with selected obstetric variables (*n*=129).

Variables	Frequency (%)	Total HRQoL score (mean±SD)	*P*
Parity			
Primiparous	70 (54.26)	65.23±12.75	0.395
Multiparous	59 (45.74)	63.17±13.02	
Number of living children			
1	78 (60.47)	65.02±12.40	0.517
2–3	51 (39.53)	63.17±13.59	
Desirability of pregnancy			
Wanted	124 (96.12)	64.91±12.42	0.040*
Unwanted	5 (3.88)	48.91±15.60	
Duration of postpartum period			
≤6 months	85 (65.89)	64.21±12.44	0.915
>6 months	44 (34.11)	64.43±13.80	
Place of delivery			
Health institution	124 (96.12)	64.10±12.61	0.487
Home	5 (3.88)	68.95±19.46	
Complications in recent delivery			
No	118 (91.47)	64.72±12.90	0.184
Yes	11 (8.53)	59.65±12.11	

* *P*≤0.05 significant, Mann–Whitney *U* test.

## Discussion

We found that the overall QoL was good in women postdelivery and was significantly associated with cesarean delivery (in the mental health subscale), occupation, family support, and pregnancy desirability. The women in whom pregnancy was desirable, employed, underwent cesarean section deliveries, and had support from spouse and family members reported a higher HRQoL score.

### Sociodemographic indicators

We found a significant association between QoL with family support, with better QoL among respondents with supportive families. The role of the spouse in general and regarding marital intimacy can also significantly impact the QoL in women postdelivery^12^. Studies by Akýn *et al*.^21^, Nohara *et al*.^32^, and Nishida *et al*.^33^ found a significant relationship between QoL and support from spouses and family members. This might be because adequate family support in activities like rest and sleep and emotional and physical care might help improve QoL. Overall, our study findings highlighted the importance of support from family and spouse for maintaining a good QoL.

### Obstetric indicators

A longitudinal study done in Spain did not find any significant difference in the QoL with different delivery modes^34^. Studies by Sadat *et al*.^20^ and Moawad *et al*.^35^ found better mental health after vaginal delivery. Davis *et al*.^36^ reported a higher QoL in women with vaginal delivery. Also, a meta-analysis by Evans *et al*.^37^ found a significantly higher QoL in women with vaginal delivery. However, similar to Torkan *et al*.^38^, we found a better mental QoL in women with cesarean delivery. This can be attributed to more care from family members following a surgical procedure, which is perceived as a complex event requiring more care. Though it is apparent that adequate care is being provided to mothers following cesarean section deliveries, there is also a need to bridge the care gap for those who had vaginal deliveries. We also found a significant relationship between the QoL of mothers postdelivery and occupation, with employed mothers having a better QoL. This is in contrast to other studies, which reported better QoL in homemakers^35^,^39^, and a decreased QoL in women who resumed their work after delivery^16^,^40^. A better QoL among employed might be due to improved access to healthcare services before, during, and after delivery^11^.

Another significant association was observed between the QoL of postnatal mothers and the desirability of pregnancy, with a better QoL in desirable pregnancies. In contrast to our findings, Gariepy *et al*.^41^ found no significant association between QoL and desirability of pregnancy. However, Akýn *et al*.^21^ and Hammoudeh *et al*.^42^ found a significant association between QoL and desirability of pregnancy. This might be because women with desirable pregnancies are mentally prepared for motherhood and its challenges, perceiving their health as good.

### Strengths and limitations

To the best of our knowledge, this is the first study conducted in Nepal using the SF-36 questionnaire in this study population. However, this study is not without limitations. Our study provides baseline data for future studies on the QoL in women postdelivery. We used nonprobability sampling because of which the results may not be generalized, and there is a possibility of information bias.

### Policy and practice implications

This study highlighted the importance of the relationship between husband and wife and emphasized the need for support from other family members. In Nepal, 77% of men and 66% of women are married^11^. Hence, awareness programs highlighting the importance of a supportive relationship can significantly impact the QoL of child-bearing women and ultimately lead to better care of the newborn. Also, there is a need to identify the reasons for a significantly lower mean HRQoL score in the mental health subscale for women having vaginal deliveries. NDHS survey of Nepal reported that 57% of women were unemployed^11^. This is alarming as our study found better HRQoL in employed women. Hence, mass employment drives should be launched to empower women, particularly in low-resource countries. This study also highlighted the importance of the desirability of pregnancy. The stakeholders should counsel the spouses regarding the significant impact of pregnancy desirability on the life of child-bearing women. Though employing women will require the stakeholders to launch various training programs after meticulous planning, other issues can be solved with minimal interventions. Preconceptional counseling of couples with emphasis on mutual support between spouses and educating couples about the importance of the desirability of pregnancy can be addressed by healthcare providers without additional costs.

### Future research

The regional variations concerning the QoL with the mode of delivery and employment reflect a need to conduct studies to identify the exact relation in different settings. Hence, more studies with larger sample sizes and randomization are warranted to identify predictors of QoL in women postdelivery.

## Conclusion

We can conclude that overall QoL was good among mothers postdelivery. The QoL of postnatal mothers following different delivery modes tends to be similar. Still, it tends to be better among postnatal mothers with cesarean section than vaginal deliveries in the mental health subscale. The QoL tends to be better among homemakers. And providing family support and making pregnancy desirable through proper counseling might help raise the QoL of postnatal mothers.

## Ethics approval

The ethical approval [Reference no. 131(6-N-E)^2^/075/076] was taken from the Institutional Review Committee, Tribhuvan University Teaching Hospital, Kathmandu, Nepal.

## Patient consent for publication

The study subjects were enrolled only after obtaining informed written consent for both participation and dissemination of results.

## Sources of funding

None.

## Author contributions

P.C.: conceptualization, data acquisition, data analysis, data interpretation, making the first draft, and critically revising the manuscript; P.P. and A.A.: data analysis, data interpretation, making the first draft, and critically revising the manuscript; A.G. and D.S.: data interpretation, making the first draft, and critically revising the manuscript. All authors approved the final version of the manuscript and are accountable for all aspects of the work.

## Conflicts of interest disclosure

There are no conflicts of interest.

## Research registration unique identifying number (UIN)

1. Name of the registry: ClinicalTrials.gov.
2. Unique identifying number or registration ID: NCT05777382.
3. Hyperlink to your specific registration (must be publicly accessible and will be checked): https://clinicaltrials.gov/ct2/show/NCT05777382.

## Guarantor

Durga Subedi.

## Data availability statement

Anonymized data will be made available on reasonable request.

## Provenance and peer review

Not commissioned, externally peer-reviewed.

## References

[1] WHO. WHOQOL – Measuring Quality of Life. Accessed 18 June 2022. https://www.who.int/tools/whoqol

[2] CDC. Health-Related Quality of Life (HRQOL). Accessed 17 June 2022. https://www.cdc.gov/hrqol/index.htm

[3] CDC. HRQOL Concepts. Accessed 18 June 2022. https://www.cdc.gov/hrqol/concept.htm

[4] Chauhan G Tadi P . Physiology, Postpartum Changes. StatPearls Publishing; 2022. Accessed 18 June 2021. https://www.ncbi.nlm.nih.gov/books/NBK555904/ 32310364

[5] Buckwalter JG Buckwalter DK Bluestein BW . Pregnancy and post partum: changes in cognition and mood. Prog Brain Res 2001;133:303–319. 11589139 10.1016/s0079-6123(01)33023-6

[6] Cho GJ Yoon HJ Kim EJ . Postpartum changes in body composition. Obesity (Silver Spring) 2011;19:2425–2428. 21701569 10.1038/oby.2011.163

[7] Meltzer-Brody S Howard LM Bergink V . Postpartum psychiatric disorders. Nat Rev Dis Primers 2018;4:18022. 29695824 10.1038/nrdp.2018.22

[8] Brockington I . Postpartum psychiatric disorders. Lancet 2004;363:303–310. 14751705 10.1016/S0140-6736(03)15390-1

[9] Amanuel T Dache A Dona A . Postpartum hemorrhage and its associated factors among women who gave birth at Yirgalem General Hospital, Sidama Regional State, Ethiopia. Health Serv Res Manag Epidemiol 2021;8:23333928211062777. 34869791 10.1177/23333928211062777 PMC8640320

[10] Hilfiker-Kleiner D Schieffer E Meyer GP . Postpartum cardiomyopathy: a cardiac emergency for gynecologists, general practitioners, internists, pulmonologists, and cardiologists. Dtsch Arztebl Int 2008;105:751–6. 19623273 10.3238/arztebl.2008.0751 PMC2696941

[11] The DHS Program. Nepal. Demographic and Health Survey 2016; 2017. Accessed 18 June 2022. https://www.dhsprogram.com/pubs/pdf/FR336/FR336.pdf

[12] Jeong YJ Nho JH Kim HY . Factors influencing quality of life in early postpartum women. Int J Environ Res Public Health 2021;18:2988. 33799474 10.3390/ijerph18062988 PMC8000893

[13] Lokubal P Calvert C Cousens S . Investigating the effect of relationship satisfaction on postpartum women’s health-related quality of life in Burkina Faso: a cross-sectional analysis. BMJ Open 2021;11:e048230. 10.1136/bmjopen-2020-048230 PMC8413953 34475164

[14] Hitimana R Lindholm L Krantz G . Health-related quality of life determinants among Rwandan women after delivery: does antenatal care utilization matter? A cross-sectional study. J Health Popul Nutr 2018;37:12. 29703248 10.1186/s41043-018-0142-4 PMC5921437

[15] Rezaei N Azadi A Zargousi R . Maternal health-related quality of life and its predicting factors in the postpartum period in Iran. Scientifica (Cairo) 2016;2016:8542147. 27022506 10.1155/2016/8542147 PMC4789062

[16] Malaju MT Alene GD Azale T . Impact of maternal morbidities on the longitudinal health-related quality of life trajectories among women who gave childbirth in four hospitals of Northwest Ethiopia: a group-based trajectory modelling study. BMJ Open 2022;12:e057012. 10.1136/bmjopen-2021-057012 PMC8921913 35288392

[17] Kavosi Z Keshtkaran A Setoodehzadeh F . A comparison of mothers’ quality of life after normal vaginal, cesarean, and water birth deliveries. Int J Community Based Nurs Midwifery 2015;3:198–204. 26171408 PMC4495327

[18] Huang K Tao F Liu L . Does delivery mode affect women’s postpartum quality of life in rural China? J Clin Nurs 2012;21:1534–1543. 22023714 10.1111/j.1365-2702.2011.03941.x

[19] Fontenele De Oliveira M Parker L Ahn H . Maternal predictors for quality of life during the postpartum in Brazilian mothers. Health 2015;07:371–380.

[20] Sadat Z Taebi M Saberi F . The relationship between mode of delivery and postpartum physical and mental health related quality of life. Iran J Nurs Midwifery Res 2013;18:499–504. 24554950 PMC3917135

[21] Akýn B Ege E Koçodlu D . Quality of life and related factors in women, aged 15-49 in the 12-month postpartum period in Turkey. J Obstet Gynaecol Res 2009;35:86–93. 19215553 10.1111/j.1447-0756.2008.00870.x

[22] Bai G Korfage IJ Mautner E . Determinants of maternal health-related quality of life after childbirth: The Generation R Study. Int J Environ Res Public Health 2019;16:3231. 31487782 10.3390/ijerph16183231 PMC6765914

[23] Alemu S Herklots T Almansa J . Mental health and quality of life of women one year after maternal near-miss in low and middle-income countries: the case of Zanzibar, Tanzania. Int J Environ Res Public Health 2020;17:9034. 33287466 10.3390/ijerph17239034 PMC7730062

[24] Khwepeya M Monsen K Kuo SY . Quality of life and the related factors in early postnatal women in Malawi. Midwifery 2020;85:102700. 32179390 10.1016/j.midw.2020.102700

[25] Ministry of Health and Population. Number of Health Facilities in Nepal – Health Emergency Operation Center. Accessed 9 June 2022. https://heoc.mohp.gov.np/service/number-of-health-facilities-in-nepal/

[26] RAND. 36-Item Short Form Survey (SF-36) | RAND. Accessed 9 June 2022. https://www.rand.org/health-care/surveys_tools/mos/36-item-short-form.html

[27] Bhandari BK Pradhan RR Pathak R . Assessment of validity of SF 36 Questionnaire using Nepali language to determine health-related quality of life in patients with chronic liver disease: a pilot study. Cureus 2018;10:e2925. 30197848 10.7759/cureus.2925 PMC6126706

[28] Mishra TA Sharma P . Health related quality of life of children with congenital heart disease attending at tertiary level hospital. J Nepal Health Res Counc 2019;17:288–292. 31735919 10.33314/jnhrc.v17i3.1789

[29] Hays RD Sherbourne CD Mazel RM . The RAND 36-Item Health Survey 1.0. Health Econ 1993;2:217–227. 8275167 10.1002/hec.4730020305

[30] RAND. 36-Item Short Form Survey (SF-36) Scoring Instructions. Accessed 9 June 2022. https://www.rand.org/health-care/surveys_tools/mos/36-item-short-form/scoring.html

[31] Mathew G Agha R Albrecht J . STROCSS 2021: strengthening the reporting of cohort, cross-sectional and case-control studies in surgery. Int J Surg 2021;96:106165. 34774726 10.1016/j.ijsu.2021.106165

[32] Nohara M Miyagi S . Family support and quality of life of pregnant women during pregnancy and after birth. Nihon Koshu Eisei Zasshi 2009;56:849–862. 20169987

[33] Nishida T Tanaka Y Sakakibara H . Factors associated with quality of life among mothers rearing 4- and 18-month old infants in Japan. Matern Child Health J 2018;22:1217–1225. 29435784 10.1007/s10995-018-2493-2

[34] Triviño-Juárez JM Romero-Ayuso D Nieto-Pereda B . Health related quality of life of women at the sixth week and sixth month postpartum by mode of birth. Women Birth 2017;30:29–39. 27353728 10.1016/j.wombi.2016.06.005

[35] Moawad S Yakout SM . Quality of life after vaginal and cesarean deliveries among a group of Egyptian women. IOSR J Nurs Health Sci (IOSR-JNHS) 2015;4:91–95.

[36] Davis DL Wu C Brown WJ . Parity and mode of birth and their relationships with quality of life: a longitudinal study. PLoS One 2022;17:e0273366. 36084030 10.1371/journal.pone.0273366 PMC9462673

[37] Evans K Fraser H Uthman O . The effect of mode of delivery on health-related quality-of-life in mothers: a systematic review and meta-analysis. BMC Pregnancy Childbirth 2022;22:149. 35193505 10.1186/s12884-022-04473-w PMC8864819

[38] Torkan B Parsay S Lamyian M . Postnatal quality of life in women after normal vaginal delivery and caesarean section. BMC Pregnancy Childbirth 2009;9:4. 19183480 10.1186/1471-2393-9-4 PMC2640344

[39] Mastaneh K Bakhteh A Rezai M . Postpartum quality of life in women after deferent modes of delivery in Kermanshah Motazedi hospital 2011–2012. J Clin Res Paramed Sci 2014;3:e82111.

[40] Chinweuba AU Okoronkwo IL Anarado AN . Differentials in health-related quality of life of employed and unemployed women with normal vaginal delivery. BMC Womens Health 2018;18:13. 29321015 10.1186/s12905-017-0481-0 PMC5764022

[41] Gariepy A Lundsberg LS Vilardo N . Pregnancy context and women’s health-related quality of life. Contraception 2017;95:491–499. 28188745 10.1016/j.contraception.2017.02.001 PMC5466832

[42] Hammoudeh W Mataria A Wick L . In search of health: quality of life among postpartum Palestinian women. Expert Rev Pharmacoecon Outcomes Res 2009;9:123–132. 19402799 10.1586/erp.09.8

